# Supplementary material for: The Immunogenicity of a Foot-and-Mouth Disease Virus Serotype O Vaccine in Commercial and Subsistence Cattle Herds in Zambia
Source: Vaccines (Basel). 2023 Dec 5;11(12):1818. doi: 10.3390/vaccines11121818 (PMC10747988; doi:10.3390/vaccines11121818)
Supplement: Supplementary file 1 [file vaccines-11-01818-s001.zip › vaccines-2715604-supplementary.pdf]

vaccines

MDPI

## Supplementary Materials

**Table S1.** The solid-phase competitive ELISA (SPCE) % inhibition results at 1:10 dilution (results for 1:30 dilution not shown) and the log reciprocal of virus neutralisation test (VNT) titres in subsistence (Rufunsa) and commercial (Chisamba) cattle herds vaccinated with either one (day 0) or two doses (day 0 and 28) of an FMDV serotype O vaccine. The SPCE and VNT results are semi-quantitative represented with a darker shade of green indicating stronger positivity and negative results indicated in grey, as explained in the legend below the Table.

| ID | Dose | Location | 1:10 SPC-ELISA (Days Post-Vaccination) | | | | | | Log VNT (Days Post-Vaccination) | | | | | | |
|---|---|---|---|---|---|---|---|---|---|---|---|---|---|---|---|
| | | | 0 | 7 | 28 | 56 | 112 | 168 | 0 | 7 | 14 | 28 | 56 | 112 | 168 |
| *RFK C2* | *Control* | *Rufunsa* | *−6.55* | *−11.70* | *−1.60* | *18.96* | *−1.30* | *−2.04* | 0.60 | 0.60 | | 1.04 | 1.20 | 1.04 | 1.20 |
| *RFK C3* | *Control* | *Rufunsa* | *−7.41* | *−13.61* | *5.55* | *0.27* | *3.10* | *7.44* | 0.60 | 1.34 | | 1.20 | 1.04 | 1.04 | 1.20 |
| RFK 32 | 1-dose | Rufunsa | −3.11 | −1.77 | 41.18 | 2.91 | 85.46 | 49.88 | 0.60 | 0.60 | | 1.65 | 1.04 | 1.51 | 1.51 |
| RFK 34 | 1-dose | Rufunsa | −1.79 | −6.41 | 17.68 | 46.55 | 10.23 | 40.14 | 0.60 | 1.20 | | 1.65 | 1.04 | 1.04 | 1.04 |
| RFK 45 | 1-dose | Rufunsa | −4.86 | 5.17 | 59.86 | 27.49 | 31.25 | 15.14 | 0.90 | 1.65 | | 1.65 | 1.65 | 0.90 | 1.34 |
| RFK 18 | 2-dose | Rufunsa | −3.42 | 0.91 | 57.52 | 87.87 | 79.91 | 83.71 | 0.60 | 1.20 | | 1.81 | 1.95 | 1.65 | 1.81 |
| RFK 21 | 2-dose | Rufunsa | −5.35 | −1.79 | 85.04 | 97.68 | 92.30 | 87.13 | 0.60 | 1.51 | | 2.25 | 2.71 | 2.25 | 1.65 |
| RFK 25/28 | 2-dose | Rufunsa | −4.40 | −7.91 | 37.96 | 97.87 | 28.28 | 5.27 | 0.60 | 1.34 | | 1.04 | 1.34 | 1.20 | 1.04 |
| RFK 43 | 2-dose | Rufunsa | −1.88 | −3.64 | −1.67 | 97.17 | 94.37 | 57.91 | 0.60 | 1.81 | | 1.65 | 2.71 | 1.51 | 1.20 |
| RFK 44 | 2-dose | Rufunsa | −2.93 | −3.90 | 48.21 | 52.35 | 85.04 | 90.01 | 1.51 | 1.20 | | 1.34 | 1.34 | 1.65 | 2.11 |
| *CSM CC1* | *Control* | *Chisamba* | | | | | | | 1.20 | | | 1.65 | 1.04 | | 0.60 |
| *CSM CC6* | *Control* | *Chisamba* | | | | | | | 0.60 | | | 1.04 | 0.60 | | 0.90 |
| *CSM CC8* | *Control* | *Chisamba* | | | | | | | 0.60 | | | 0.60 | 0.60 | | 0.60 |
| *CSM CC9* | *Control* | *Chisamba* | | | | | | | 0.90 | | | 1.04 | 0.90 | | 1.04 |
| CSM C1 | 1-dose | Chisamba | 3.90 | | 3.81 | 11.92 | 8.19 | −4.76 | 0.60 | | | 1.04 | 0.60 | 0.60 | 0.60 |
| CSM C2 | 1-dose | Chisamba | −0.56 | | −0.17 | 34.08 | 30.78 | 18.23 | 0.60 | | | 1.04 | 0.60 | 0.60 | 1.04 |
| CSM C4 | 1-dose | Chisamba | −1.42 | | −3.95 | 31.06 | 2.65 | 10.46 | 0.60 | | | 1.04 | 1.04 | 1.04 | 0.60 |
| CSM C5 | 1-dose | Chisamba | 3.01 | | 68.64 | 57.65 | 8.04 | 41.47 | 0.60 | | | 1.34 | 1.20 | 1.04 | 0.90 |
| CSM C6 | 1-dose | Chisamba | 2.44 | | 38.08 | 6.26 | −3.50 | 17.20 | 1.65 | | | 1.04 | 1.04 | 0.60 | 1.20 |
| CSM C7 | 1-dose | Chisamba | 2.00 | | 1.42 | 31.21 | 9.86 | 2.01 | 0.60 | | | 1.51 | 0.90 | 0.60 | 0.60 |
| CSM C8 | 1-dose | Chisamba | 6.64 | | 28.11 | 90.55 | 44.85 | 65.51 | 0.60 | | | 1.81 | 1.51 | 1.65 | 1.20 |
| CSM C9 | 1-dose | Chisamba | 6.43 | | 2.81 | 2.16 | −2.64 | −5.26 | 0.60 | | | 1.04 | 0.60 | 0.60 | 0.90 |
| CSM C10 | 1-dose | Chisamba | 4.23 | | −1.28 | 15.49 | 28.54 | 19.41 | 1.20 | | | 1.51 | 1.04 | 1.04 | 1.04 |
| CSM 4 | 2-dose | Chisamba | −1.15 | | 18.82 | 88.21 | 23.34 | 2.07 | 0.60 | 0.60 | 0.90 | 1.04 | 1.65 | 1.04 | 0.60 |
| CSM 8 | 2-dose | Chisamba | −1.66 | | 43.20 | 74.15 | 59.93 | 66.79 | 0.60 | 1.04 | 1.81 | 1.95 | 1.51 | 1.81 | 1.81 |
| CMS 14 | 2-dose | Chisamba | 2.47 | | 14.22 | 90.00 | 41.71 | 65.93 | 0.60 | 1.04 | 1.81 | 1.20 | 1.95 | 1.34 | 1.20 |
| CSM 15 | 2-dose | Chisamba | 1.29 | | −1.54 | 90.64 | 50.88 | 34.48 | 1.04 | 1.04 | 1.34 | 1.04 | 2.11 | 1.04 | 1.04 |
| CSM 16 | 2-dose | Chisamba | 3.75 | | 2.00 | 92.49 | 55.59 | 42.80 | 0.60 | 0.90 | 1.34 | 1.34 | 1.95 | 1.34 | 1.65 |
| CSM 17 | 2-dose | Chisamba | 0.19 | | 3.63 | 96.34 | 90.46 | 86.55 | 0.90 | 1.04 | 1.04 | 1.34 | 2.71 | 2.25 | 1.65 |
| CSM 22 | 2-dose | Chisamba | 0.27 | | 3.40 | 97.35 | 38.69 | 40.04 | 0.90 | 1.04 | 1.34 | 1.51 | 2.55 | 1.95 | 2.55 |
| CSM 24 | 2-dose | Chisamba | 0.54 | | 28.19 | 98.18 | 95.75 | 97.70 | 1.04 | 0.60 | 1.04 | 1.04 | 1.34 | 0.90 | 1.04 |
| CSM 25 | 2-dose | Chisamba | 3.90 | | 6.37 | 86.03 | 88.67 | 87.45 | 0.90 | 0.90 | 1.20 | 1.20 | 2.41 | 1.65 | 1.34 |

**Table S1.** Legend.

| | SPCE | | VNT |
|---|---|---|---|
| (grey) | <70 (1:10) | (grey) | ≤1.04 |
| (light green) | ≥70 (1:10); <80 (1:10) | (light green) | >1.04; <1.51 |
| (bright green) | ≥80 (1:10); <50 (1:30) | (bright green) | =1.51 |
| (dark green) | ≥80 (1:10); ≥50 and <80 (1:30) | (dark green) | >1.51 |
| (dark grey) | ≥80 (1:10); ≥80 (1:30) | | |
